# CT imaging features associated with recurrence in non-small cell lung cancer patients after stereotactic body radiotherapy

**DOI:** 10.1186/s13014-017-0892-y

**Published:** 2017-09-25

**Authors:** Qian Li, Jongphil Kim, Yoganand Balagurunathan, Jin Qi, Ying Liu, Kujtim Latifi, Eduardo G. Moros, Matthew B. Schabath, Zhaoxiang Ye, Robert J. Gillies, Thomas J. Dilling

**Affiliations:** 1 Department of Radiology, Tianjin Medical University Cancer Institute and Hospital, National Clinical Research Center for Cancer, Key Laboratory of Cancer Prevention and Therapy, Tianjin’s Clinical Research Center for Cancer, Huan-Hu-Xi Road, Ti-Yuan-Bei, He Xi District, Tianjin, 300060 China; 2 Department of Cancer Imaging and Metabolism, H. Lee Moffitt Cancer Center and Research Institute, Tampa, FL USA; 3 Department of Biostatistics and Bioinformatics, H. Lee Moffitt Cancer Center and Research Institute, Tampa, FL USA; 4 Department of Radiation Oncology, H. Lee Moffitt Cancer Center and Research Institute, 12902 Magnolia Drive, Tampa, FL 33612 USA; 5 Department of Cancer Epidemiology, H. Lee Moffitt Cancer Center & Research Institute, Tampa, FL USA

**Keywords:** Stereotactic body radiotherapy (SBRT), Computed tomography, Survival, Radiomics, Semantics, Image features

## Abstract

**Background:**

Predicting recurrence after stereotactic body radiotherapy (SBRT) in non-small cell lung cancer (NSCLC) patients is problematic, but critical for the decision of following treatment. This study aims to investigate the association of imaging features derived from the first follow-up computed tomography (CT) on lung cancer patient outcomes following SBRT, and identify patients at high risk of recurrence.

**Methods:**

Fifty nine biopsy-proven non-small cell lung cancer patients were qualified for this study. The first follow-up CTs were performed about 3 months after SBRT (median time: 91 days). Imaging features included 34 manually scored radiological features (semantics) describing the lesion, lung and thorax and 219 quantitative imaging features (radiomics) extracted automatically after delineation of the lesion. Cox proportional hazard models and Harrel’s C-index were used to explore predictors of overall survival (OS), recurrence-free survival (RFS), and loco-regional recurrence-free survival (LR-RFS). Five-fold cross validation was performed on the final prognostic model.

**Results:**

The median follow-up time was 42 months. The model for OS contained Eastern Cooperative Oncology Group (ECOG) performance status (HR = 3.13, 95% CI: 1.17–8.41), vascular involvement (HR = 3.21, 95% CI: 1.29–8.03), lymphadenopathy (HR = 3.59, 95% CI: 1.58–8.16) and the 1st principle component of radiomic features (HR = 1.24, 95% CI: 1.02–1.51). The model for RFS contained vascular involvement (HR = 3.06, 95% CI: 1.40–6.70), vessel attachment (HR = 3.46, 95% CI: 1.65–7.25), pleural retraction (HR = 3.24, 95% CI: 1.41–7.42), lymphadenopathy (HR = 6.41, 95% CI: 2.58–15.90) and relative enhancement (HR = 1.40, 95% CI: 1.00–1.96). The model for LR-RFS contained vascular involvement (HR = 4.96, 95% CI: 2.23–11.03), lymphadenopathy (HR = 2.64, 95% CI: 1.19–5.82), circularity (F13, HR = 1.60, 95% CI: 1.10–2.32) and 3D Laws feature (F92, HR = 1.96, 95% CI: 1.35–2.83). Five-fold cross-validated the areas under the receiver operating characteristic curves (AUC) of these three models were all above 0.8.

**Conclusions:**

Our analysis reveals disease progression could be prognosticated as early as 3 months after SBRT using CT imaging features, and these features would be helpful in clinical decision-making.

**Electronic supplementary material:**

The online version of this article (10.1186/s13014-017-0892-y) contains supplementary material, which is available to authorized users.

## Introduction

Stereotactic body radiotherapy (SBRT) is a guideline-recommended treatment of choice for patients with early stage non-small cell lung cancer (NSCLC) who are inoperable or do not accept the risk of surgery [1]. SBRT delivers high doses of radiation in five or fewer fractions to the targeted area with high local control and sparing normal tissues. There have been a few studies showing improved overall survival in patients treated with SBRT [2] and it has replaced conventionally fractionated radiotherapy as standard of care in treatment of stage I disease.

One of the most serious complications of SBRT is radiation induced lung injury (RILI). Acute radiation pneumonitis is generally seen in approximately 10% of patients and fibrosis in most of the cases [3], which makes the follow-up response assessment especially difficult. Although most mass-like consolidations in RILI decrease in size with time, there are also RILI cases with transient size increases. Conversely, patients with recurrence may show temporary size decreases [4]. It has been shown in several studies that size alone is not a reliable criterion until 12 months or more after SBRT [4, 5]. Early prediction of recurrence is critical as alternative treatments such as salvage surgery or systemic therapy may be still available for many of these patients. Several studies have focused on finding early imaging markers to predict disease progression. Studies on pre-treatment positron emission tomography - computed tomography (PET-CT) still remain controversial about the predictive power of maximum standardized uptake value (SUVmax) [6–14]. A recent study on post-treatment PET showed that future local recurrence could be predicted 3 months after SBRT [15]. However, CT is the standard modality for imaging follow-up for SBRT patients, and FDG-PET is generally only performed when recurrence is suspected. Previous studies have identified a few CT image features related to recurrence [5, 16], such as sequentially enlarging mass-like lesion, opacity enlargement after 12 months, filling-in of air bronchograms, bulging margins, disappearance of the linear margin, development of ipsilateral pleural effusion, or subsequent lymph node enlargement. The study conducted by Huang et al. [17] showed these features were prognostic in univariate analysis, though the best one was still opacity enlargement after 12 months. Another study [18] found that density increase, filling-in of air bronchograms or new mass or effusion were not reliable features.

Obviously, the above features are not sufficient to predict recurrence, and more reliable features are needed. Recently, “radiomic” approaches have been shown to better describe a given region of interest and furthermore, these radiomic features are also predictive of patient outcome [19]. Mattonen et al. [20] demonstrated that texture measures of the ground glass opacity (GGO) appearance following SBRT could predict recurrence in individual patients within 5 months of SBRT treatment. The study showed appropriate use of radiomic descriptors for outcome prediction, although the definition of the GGO area was relatively subjective. Additionally, radiomics focuses only on the delineated area. As such, an evaluation of the lung field may provide more information about recurrence, and a general evaluation of the patient's status may provide more information about the health, possibly related to outcome. In this study, we systemically scored the radiological features related to the lesion, the lung and the thorax, and extracted the radiomic features of the solid tumor. We then built a model by combining these features to prognosticate recurrence.

## Patients and methods

### Patients

This retrospective study was approved by our institutional review board (#105996) and informed consent was waived. We identified 59 patients that were treated with SBRT between January 2009 and July 2013. Inclusion criteria allowed patients with primary lung cancers confirmed by biopsy and without prior lung radiation or prior lung tumor history, TNM stage ≤ IIA (node negative), and contrast-enhanced CT images with both mediastinum and lung window settings. The slice thickness was required to be ≤3 mm. Patients excluded were those with more than one lung tumor or other concurrent tumors in other sites, those that received other treatment before SBRT, and those whose tumors could not be identified after SBRT or lacked data to confirm recurrence. Clinical information collected at the time of treatment included age, gender, clinical TNM stage, clinical T stage, smoking status, pack-years smoking history, O_2_ dependence or not, Eastern Cooperative Oncology Group (ECOG) performance status and Charlson comorbidity index (CCI).

A regimen of 50 Gy in 5 fractions was the standard treatment option and was administered in 54 patients (91.5%), while three patients (5.1%) received 48 Gy in 4 fractions and another 2 patients were treated with 60 Gy in 8 or 5 fractions.

The heterogeneity corrected collapsed cone convolution (CCC) algorithm was used for treatment planning. Either 3D conformal or volumetric arc therapy (VMAT) techniques were used, with photon beam energies ranging from 6 to 15 MV. The patients were treated on a Trilogy or a True Beam medical linear accelerator (Varian Medical Systems, Palo Alto, CA) equipped with a 120-leaf Millennium multi-leaf collimator (5-mm leaves in the central portion of the field). Daily image guidance was provided by cone beam computed tomography (CBCT), with alignment to the visible tumor on the planning CT scan. Dose voxel size was kept at 2 mm.

### Patient follow-up and outcomes

Follow-up evaluations were based on CT images and clinical examination. They were performed every 3 months in the first 2 years after SBRT, then every 4–6 months for the following 3 years, and annually thereafter. An ^18^F–FDG-PET/CT scan was recommended when recurrence or metastasis was suspected.

Local recurrence was defined as progression of the original primary lesion or new tumors in the same lobe as the primary tumor. Regional recurrence was defined as hilar or mediastinal lymph node metastasis. Distant metastasis was defined as tumors in other lobes of the lung or outside the lung. Recurrence was confirmed by biopsy, PET/CT, or CT images at follow-up. The recurrence date was recorded as the date of first CT or PET/CT scan that showed signs of progression.

Three clinical end points were analyzed: overall survival (OS), recurrence-free survival (RFS) and loco-regional recurrence free survival (LR-RFS). OS was calculated from the start date of SBRT to the last follow-up date (for censored cases) or date of death. RFS was calculated from the start date of SBRT to the date of local, regional or distant metastasis, or the date of death, or censored at the last follow-up date. The LR-RFS time was calculated from the start date of SBRT to the date of local or regional recurrence, or the date of death, or censored at the last follow-up date.

### First follow-up CT scan protocol and image assessment

The first follow-up CT scan was performed 1 to 3 months after SBRT (33–112 days, median time: 91 days) using one of the following multi-detector CT scanners: Light Speed pro 32 (GE Medical System), Sensation 16, Sensation 40, or Sensation 64 (Siemens Healthcare, Germany). Examinations were performed after intravenous administration of contrast material (1.3–1.5 ml per kilogram of body weight) at the rate of 2 mL/s. Scanning parameters were as follows: 120 kVp with tube current adjusted automatically, and 2.5 mm or 3 mm reconstruction thickness.

CT images were reviewed by two radiologists using both mediastinal (width, 350 HU; level, 40 HU) and lung (width, 1500 HU; level, −600 HU) window settings. Both of them were blind to clinical and histologic findings. A total of 34 semantic features were developed. Some of these features have been reported in our previous study [21] and were found to be associated with epidermal growth factor receptor mutation status in patients with lung adenocarcinoma. In this study, more features were evaluated, including features about the lesion, the lung and the thorax (detailed descriptions in Additional file 1: Table S1). Intra-class correlation coefficient (ICC) and (weighted) Kappa index were used to evaluate the concordance between radiologists. Final score of these features were based on the consensus between the two radiologists (average value for continuous variables).

The radiomic features were extracted using the Definiens Developer® (Munich Germany) image analysis software [22, 23]. At first, pre-processing was done automatically to segment the lung, body and background. In some cases, segmentations were edited by the radiologist so as to include proper segmentation of juxtapleural lesions. Then, a click and grow segmentation method developed by our group was used to contour the lesion [24]. The delineated region was checked and in some cases further corrected by a radiologist. Finally, in these regions of interest, 219 three dimensional (3D) image features [25] were computed that can be broadly categorized into first order statistics (such as histogram features), second order statistics (such as features based on the gray level co-occurrence matrix) and higher order statistics (such as wavelet decomposition).

### Statistical analysis

Seven semantic features and one radiomic feature, including distribution, fissure attachment, attenuation, calcification, pleural effusion of non-tumor side, new/enlarging nodules in primary tumor lobe, new/enlarging nodules in non-tumor lobe and MacSpic_NumberOf (F7), were excluded from further analysis because most patients were in the same category. To address the issue of collinearity, Pearson’s correlation analysis was performed for the remaining 218 radiomic features to eliminate redundant features. This eliminated 52 features that were highly dependent on one another (the absolute value of Pearson’s correlation >0.95). Ultimately, this methodology resulted in 27 semantic features and 166 radiomic features that were used for the analysis.

The statistical analyses were performed using SAS software (version 9.4, Cary, NC) and the computed *P* - values were two-sided. Cox proportional hazards models were used to explore the association between clinical and imaging features with OS, RFS and LR-RFS. Clinical and semantic features with *p*-value of <0.1 and radiomic features with *q*-value (false discovery rate adjusted *p* value) of <0.1 in univariate model were incorporated into the initial multivariate model. The final model was selected by either stepwise selection or backward elimination method (if different models were built using these two methods, the one with higher concordance would be selected). The hazard ratio (HR) and 95% confidence interval (CI) were calculated.

As part of our analysis, we utilized principle component analysis (PCA) [26] to reduce the dimensionality and explore abstractive patterns. The goal is to extract the important information from the data and to express this information as a set of new orthogonal variables called principal components, which are linear combinations of the original variables; the first principle component (PC1) describes the most variance in the data and is considered the most descriptive. The second component (PC2) is computed under the constraint of being orthogonal to the first component. The other components are computed likewise (PCn, *n* = 1, 2, 3 …). The values of these new variables can be interpreted geometrically as the projections of the observations onto the principal components.

The final multivariate prognostic model was built by combining clinical with imaging features (semantics and radiomics, including PC1 and PC2). Harrell’s C-index [27] was computed to describe the performance of each prognostic model. The model with highest Harrell’s C-index was selected as the best prognostic model, and the risk scores of OS, RFS and LR-RFS based on these models were developed accordingly. Patients were dichotomized into low and high groups on the basis of their median risk score*.* The Kaplan-Meier method was used to estimate survival curves.

Five-fold cross-validated area under receiver operating characteristic curves (AUC) with 100 replications of each model were computed after dichotomizing these patients into short- and long-term survival group according to their survival status at 24 months (Additional file 1: Table S5). The follow-up time of three surviving patients was less than 24 months in calculating OS, and therefore they were excluded from validation analysis.

## Results

The demographic information is provided in Table 1. Among the 59 patients, 23 (39%) were females and 36 (61%) were males (median age, 73 years). The median follow-up time was 42 months (range: 6.5–67.3 months). At the end of this study, a total of 33 patients developed recurrence or metastasis (Additional file 1: Table S2), and 24 of them occurred within 2 years. The two-year OS, regional failure and distant failure rate were 69.95%, 8.47% and 25.42%, respectively. The “local recurrence” here was defined as intra-lobar, which is about 11.86% and 8.47% of them was in-field failure.

**Table 1:** Clinical and treatment characteristics of NSCLC patients treated with SBRT

Clinical features	Level	n	%
Gender	Female	23	39.0
	Male	36	61.0
Age, median (range) (years)	73 (48–91)		
Age	≤67	16	27.1
	68–80	28	47.5
	≥81	15	25.4
TNM stage	IA	42	71.2
	IB	16	27.1
	IIA	1	1.7
T stage	1A or 1B	42	71.2
	2A or 2B	17	28.8
Smoker^a^	No	42	72.4
	Yes	16	27.6
Pack-years smoking^a^	median (range)	58 (15–200)	
	≤40	14	25.0
	41–79	27	48.2
	≥80	15	26.8
O_2_ dependence	No	45	79.0
	Yes	12	21.1
ECOG	0 or 1	49	84.5
	2 or 3	9	15.5
CCI, median (range)	6 (3–12)		
CCI	≤4	18	30.5
	5–7	28	47.5
	≥8	13	22.0
Dose/Fx	7.5	1	1.7
	10	54	91.5
	12	4	6.8
Pathology	adenocarcinoma	26	44.1
	squamous cell carcinoma	21	35.6
	NSCLC	10	16.9
	large cell carcinoma	2	3.4

^a^The pack-years smoking history could not be found in 2 cases and in one additional patient the smoking history was not noted

### Prognostic clinical features

As expected, T stage and ECOG performance status were associated (*P* < 0.05) with OS and LR-RFS both in univariate and multivariate analysis, while only T stage (*P* = 0.01, HR = 2.26, 95% CI: 1.19–4.30) was independently related with RFS.

### Prognostic semantic features

According to Viera’s criteria [28], the agreements between two readers on scoring of categorical variables were substantial or almost perfect, and the Kappa value ranged from 0.68–1.0 (Additional file 1: Table S3). The ICCs for long and short axial diameter, relative enhancement were 0.95 (95% CI: 0.92–0.97), 0.92 (95% CI: 0.87–0.95), and 0.71 (95% CI: 0.54–0.82), respectively.

In univariate analysis, long- and short-axis diameter, border definition, vascular involvement, lymphadenopathy, pleural effusion of tumor side and relative enhancement were prognostic (*P* < 0.05) of all three endpoints. Lobe location and pleural attachment was associated with OS and RFS (*P* < 0.1); Spiculation was prognostic for RFS and LR-RFS (*P* < 0.05). Vessel attachment (*P* = 0.06), pleural retraction (*P* = 0.05) and thickened adjacent bronchovascular bundle (*P* = 0.03) were prognostic of RFS.

**Fig. 1:**
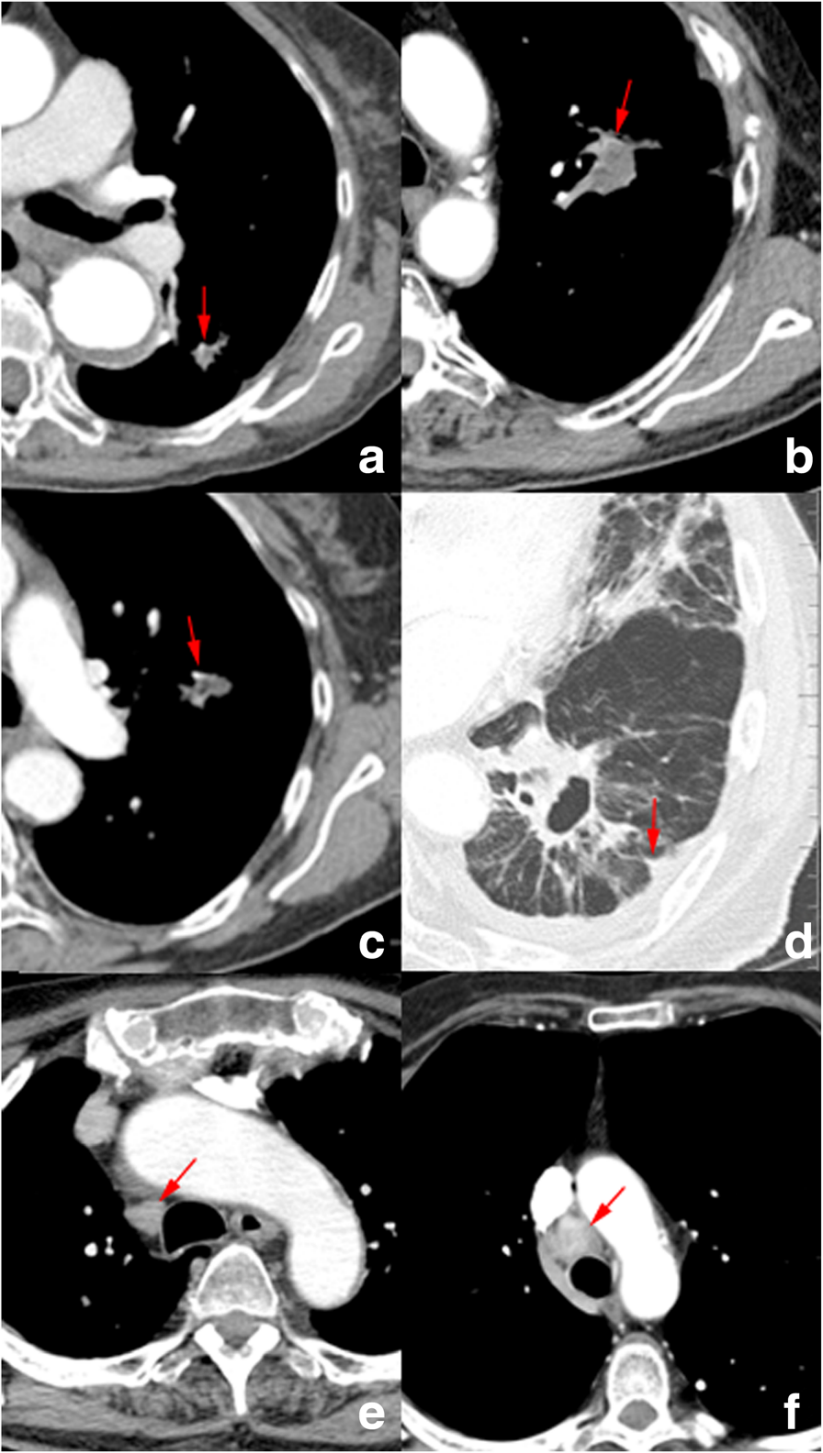
Examples of CT images showing typical semantic features (**a** - **b**: vascular involvement; **c**: vessel attachment; **d**: pleural retraction: **e**: benign lymphadenopathy, **f**: malignant lymphadenopathy)

In multivariate analysis, vascular involvement and lymphadenopathy at first follow-up scan (Fig. 1) remained independently associated with shorter OS, RFS and LR-RFS. Vessel attachment, pleural retraction and relative enhancement were additional independent indicators of LR-RFS. It should be pointed out that the relative enhancement here was measured by using the artery on the same slice as reference (Additional file 1: Table S1), as the pre-contrast images were not available for these patients.

### Prognostic radiomic features

In univariate analysis, 7 radiomic features were found to be significantly prognostic of OS, 12 of RFS and 15 of LR-RFS (Additional file 1: Table S4). However, in multivariate analysis, only circularity (F13) remained correlated with OS (*P* < 0.00, HR = 2.09, 95% CI: 1.40–3.12). Radius of smallest enclosing ellipse (F29, *P* = 0.00, HR = 1.59, 95% CI: 1.18–2.15) and 3D Wavelet decomposition P1 L2 C3 Layer 1 (F214, *P* = 0.02, HR = 1.49, 95% CI: 1.07–2.08) were independently prognostic of RFS. Circularity (F13, *P* < 0.00, HR = 2.10, 95% CI: 1.47–2.99) and 3D Laws features L5 L5 S5 Layer 1 (F92, *P* = 0.00, HR = 1.92, 95% CI: 1.34–2.75) were independently prognostic of LR-RFS.

### Prognostic model and prognostic index

The final prognostic models (Table 2) were built by combing the clinical and imaging features. The Harrell’s C indexes of the imaging models were higher than those of the clinical models (Table 3). The final prognostic model of OS involved both clinical and imaging features, and it was superior to clinical or imaging features alone. While the best prognostic model for RFS and LR-RFS included only imaging features. After 5-fold cross validation was performed, the AUC value of the OS, RFS and LR-RFS models were 0.81, 0.80 and 0.80, respectively (Table 2). The risk score of OS (OSRS), RFS (RFRS) and LR-RFS (LRRS) were as follows:

$$ \mathbf{OSRS}=0.21391\times \left(\mathrm{PC}1\ \mathrm{of}\ \mathrm{OS}\hbox{--} \mathrm{mean}\ \left(\mathrm{PC}1\ \mathrm{of}\ \mathrm{OS}\right)\right)+1.14224\times {\mathrm{I}}_{\left\{\mathrm{ECOG} =^{\hbox{'}}2\ \mathrm{or}\ {3}^{\hbox{'}}\right\}}+1.1674\times {\mathrm{I}}_{\left\{\mathrm{vessel}\  \mathrm{involvement}=1\right\}}+1.27826\times {\mathrm{I}}_{\left\{\mathrm{lymphadenopathy}=1\right\}} $$

$$ {\displaystyle \begin{array}{l}\mathbf{RFRS}=1.11755\times {\mathrm{I}}_{\left\{\mathrm{vascular}\  \mathrm{involvement}=1\right\}}+1.24121\times {\mathrm{I}}_{\left\{\mathrm{vessel}\  \mathrm{attachment}=1\right\}}+1.17506\times {\mathrm{I}}_{\left\{\mathrm{pleural}\  \mathrm{retraction}=1\right\}}+1.85753\times {\mathrm{I}}_{\left\{\mathrm{lymphadenopathy}=1\right\}}+0.33705\times \\ {}\left(\mathrm{relative}\  \mathrm{enhancement}\hbox{--} \mathrm{mean}\ \left(\mathrm{relative}\  \mathrm{enhancement}\right)\right)/\mathrm{SD}\ \left(\mathrm{relative}\  \mathrm{enhancement}\right)\end{array}} $$

$$ {\displaystyle \begin{array}{l}\mathbf{LRRS}=0.46669\times \left(\mathrm{F}13\hbox{--} \mathrm{mean}\ \left(\mathrm{F}13\right)\right)/\mathrm{SD}\ \left(\mathrm{F}13\right)+0.6715\times \left(\mathrm{F}92\hbox{--} \mathrm{mean}\ \left(\mathrm{F}92\right)\right)/\mathrm{SD}\ \left(\mathrm{F}92\right)\\ {}+1.60193\times {\mathrm{I}}_{\left\{\mathrm{vascular}\  \mathrm{involvement}=1\right\}}+0.96896\times {\mathrm{I}}_{\left\{\mathrm{lymphadenopathy}=1\right\}}\end{array}} $$

**Table 2:** The features involved in prognostic models of OS, RFS and LR-RFS

	Features	Level	*p*-value	Hazard Ratio			validation	
				Point	95% CI		Logistic regression	5-fold cross validation
					Lower	Upper	AUC (95% CI)	AUC (95% CI)
OS	ECOG (ref = 0 or 1)	2 or 3	0.02	3.13	1.17	8.41	0.88 (0.78–0.97)	0.81 (0.80–0.82)
	Vascular involvement (ref = 0)	1	0.01	3.21	1.29	8.03		
	Lymphadenopathy (ref = 0)	1	0.00	3.59	1.58	8.16		
	PC1^a^		0.04	1.24	1.02	1.51		
RFS	Vascular involvement (ref = 0)	1	0.01	3.06	1.40	6.70	0.86 (0.76–0.96)	0.80 (0.792–0.81)
	Vascular attachment (ref = 0)	1	0.00	3.46	1.65	7.25		
	Pleural retraction (ref = 0)	1	0.01	3.24	1.41	7.42		
	Lymphadenopathy (ref = 0)	1	<.00	6.41	2.58	15.90		
	Relative enhancement^a^		0.05	1.40	1.00	1.96		
LR-RFS	F13 (9b_3D_Circularity)^a^		0.02	1.60	1.10	2.32	0.85 (0.74–0.95)	0.80 (0.78–0.81)
	F92 (3D Laws features L5 L5 S5 Layer 1)^a^		0.00	1.96	1.35	2.83		
	Vascular involvement (ref = 0)	1	<.00	4.96	2.23	11.03		
	Lymphadenopathy (ref = 0)	1	0.02	2.64	1.19	5.82		

^a^: per 1standard deviation (SD) increase

ECOG, Eastern cooperative oncology group; PC1: the 1st principle component

**Table 3:** Harrell’s C-index for prognostic models of OS, RFS and LR-RFS

	Models	Features	Harrell’s C index		
			Point	95% CI	
				Lower	Upper
OS	Clinical features	T-stage & ECOG^a^	0.64	0.54	0.74
	Imaging features	Vascular involvement, lymphadenopathy, F13^a^	0.76	0.67	0.84
	Clinic & imaging features	ECOG, vascular involvement, lymphadenopathy, PC1	0.78	0.71	0.86
RFS	Clinical feature	T-stage	0.61	0.54	0.68
	Imaging features	Vascular involvement, vascular attachment, pleural retraction, lymphadenopathy, relative enhancement	0.77	0.70	0.84
	Clinic & imaging features	Vascular involvement, vascular attachment, pleural retraction, lymphadenopathy, relative enhancement	0.77	0.70	0.84
LR-RFS	Clinical features	T-stage & ECOG^a^	0.62	0.54	0.70
	Imaging features	Vascular involvement, lymphadenopathy, F13^a^, F92^a^	0.78	0.71	0.85
	Clinic & imaging features	Vascular involvement, lymphadenopathy, F13^a^, F92^a^	0.78	0.71	0.85

^a^ECOG, Eastern cooperative oncology group; PC1: the 1st principle component; F13: 9b_3D_Circularity; F92: 3D Laws features L5 L5 S5 Layer 1

I _{.}_ is the identity function.

**Fig. 2:**
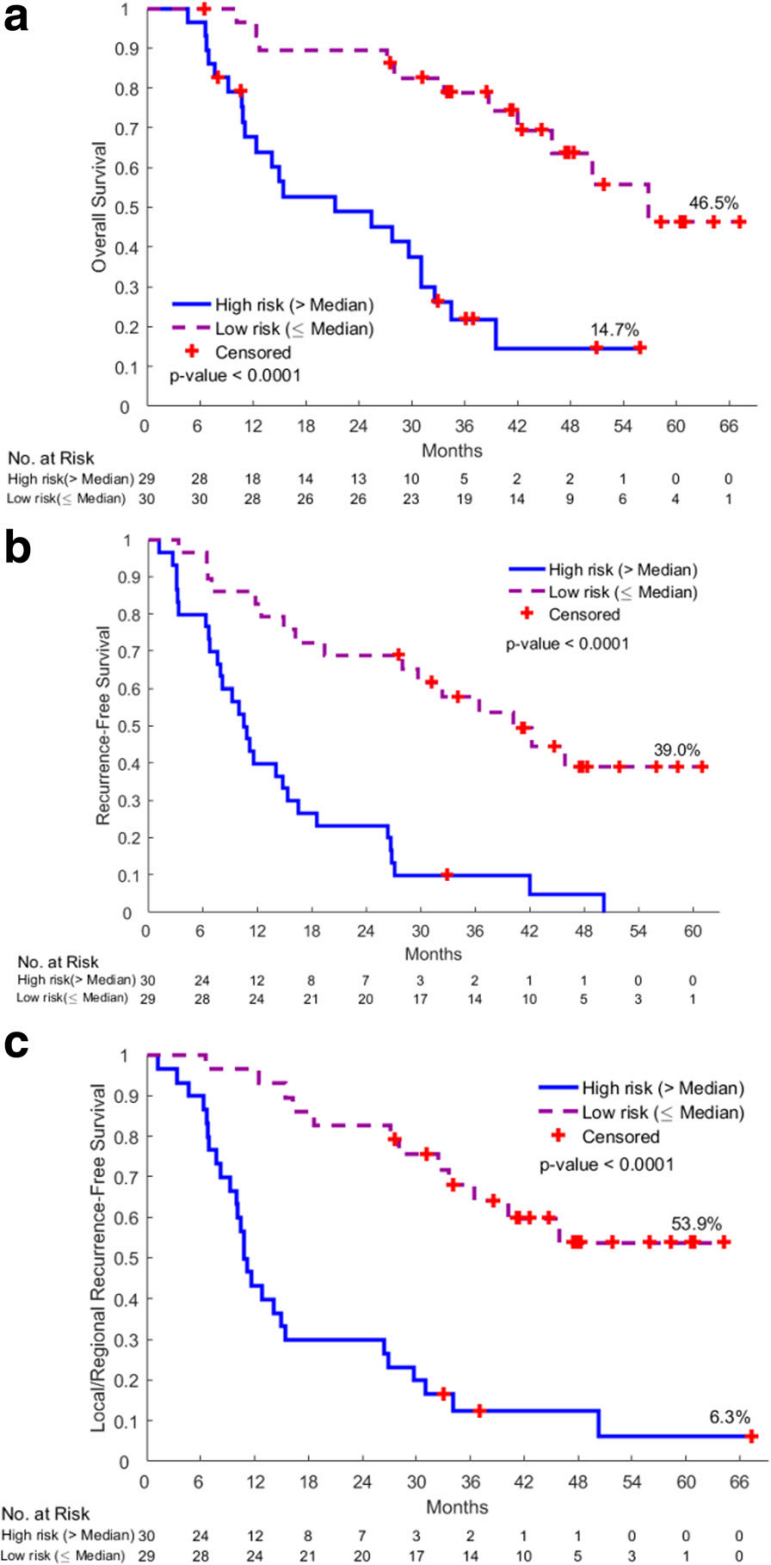
Kaplan-Meier Plots of overall survival (**a**), recurrence free survival (**b**) and loco-regional recurrence free survival (**c**) according to the prognostic risk scores

The performance of each risk score is shown in Fig. 2. In these figures, the patients were divided into two groups, depending whether their index scores were greater than the median score or less than/equal to the median score. The resultant Kaplan-Meier plots for the two groups were highly statistically divergent in regard to OS, RFS and LR-RFS.

## Discussion

Based on a comprehensive analysis of CT images, we found both semantic and radiomic features were significantly associated with patient outcomes following SBRT. As such, the analysis described has the potential to predict disease recurrence as early as 3 months post-SBRT, and therefore it would be of great help for clinical decision making.

The Response Evaluation Criteria in Solid Tumor (RECIST) guideline is developed for the assessment of treatment outcome and it is mainly based on the percentage change in tumor size. For example, tumor shrinkage of more than 30% is considered to be partial response. However, in our study, the percentage of shrinkage was not an independent prognostic factor of survival and the actual tumor size after SBRT was only significant in univariate analysis. Mattonen et al. [20] reported that the overall accuracy of RECIST for predicting tumor recurrence at 2–5 months post-SBRT was 52.2% (i.e., equivalent to chance). This suggests that size-related features are not suitable for early prediction of SBRT outcome, and it may not be reliable until 12 months after SBRT [4, 5]. Therefore, we should take caution when using RECIST especially in the early days after treatment.

The T stage of the tumor before therapy was independently associated with OS, RFS and LR-RFS, which implies that pre-treatment tumor size is predictive of ultimate outcome. This is not surprising, given that T-stage (T1a versus T1b versus T2b) relates to overall stage (IA, IB, or IIA), which predicts various outcomes. Additionally, total dose was not considered as prognostic factor in our analysis, as 91.5% of patients were treated with 50 Gy in 5 fractions. In the future, if we had more patients involved, we would categorize the patients according to the dose/fractions. It would be helpful to investigate the influence of dose on outcomes.

Blood supply is essential for tumor growth and metastasis. Tumors gain access to blood supply either by invading existing vessels or by generating new vessels via angiogenesis. Tumor cells could penetrate these vessels and escape from the primary site to distant organs [29]. Consequently, vessel invasion has been felt by some to be an unfavorable prognostic factor [30, 31], even in early stage NSCLC [32]. One of the advantages of contrast-enhanced follow-up images was optimal display of the vascular structures, such as vessel involvement or attachment. The degree of tumor enhancement has been demonstrated [33] to be positively correlated with tumor vascular density and vascular endothelial growth factor (VEGF) expression, and suggested to reflect the number of small tumoral vessels [34]. Thus, high levels of enhancement after SBRT suggests highly vascularized tumor, and is related with poor outcome. Similar findings have also been reported in chemotherapy or chemoradiotherapy patients [35]. Relative enhancement, together with vascular involvement and vessel attachment, may just reflect angiogenesis and vessel invasion, respectively.

Previously identified high-risk CT features [16] were also found to be prognostic in our analysis, such as pleural effusion and poorly defined borders. Whereas most of these features were significant in univariate analysis, only lymph node enlargement was still significant in multivariate analysis. Wang et al. [36] also reported that lymphadenopathy was significantly associated with an increased risk of death in adenocarcinoma. Lymphadenopathy in CT images is usually defined as lymph nodes larger than 10 mm in short axis [37]. However, according to this criterion, the accuracy of predicting malignancy was only about 63% [38]. Therefore, lymphadenopathy does not definitely mean lymph node metastasis. Figure 1e and f both show enlarged lymph nodes in the follow-up CT scans, but in one case the lymph node biopsy was negative and in the other it was positive. It would appear that lymphadenopathy secondary to *any* cause is predictive of recurrence in our model, and not necessarily specifically as a harbinger of regional lymph node spread. Pleural retraction was another prognostic factor of RFS. It is usually taken as a sign of malignancy, and may be related to visceral pleural surface invasion [39]. It should be noted that pleural retraction after SBRT can result from radiation fibrosis (Fig. 1d).

Radiomics analysis explores tumor heterogeneity and provides large number of quantitative descriptors. In this study, we extracted 219 features from the solid tumor of each patient visible in the first post-SBRT CT scan, and these features have been tested in our previous study [25]. Semantically or radiologically, the shape of a tumor is defined as either round/oval or (somewhat) irregular. While in radiomics tumor shape can be expressed by multiple continuous variables such as circularity (F13) and radius of smallest enclosing ellipse (F29) which describe how spherical or elliptical a tumor is, respectively. The Laws features (F92) and wavelet decompositions (F214) are higher order statistics obtained by applying filters to the image. The “laws” features [40] were constructed from a set of five one-dimensional filters, each designed to reflect a different type of structure in the image. Wavelet features are kernel-based functions that decompose the image (3D) into orthogonal components. These radiomic features were either associated with worse OS, or RFS, or LR-RFS. It means that if the tumor is less spherical or elliptical, or if the tumor is more heterogeneous in shape and/or density, it tended to progress. To date, the radiomic and semantic features are complementary to each other, and neither one of them could be replaced by the other. Thus, combining them together is a better approach.

The results discussed above could have great clinical import. One could utilize these various features to predict which patients are at increased risk of recurrence and escalate their therapy accordingly to improve their ultimate outcomes. For instance, if a patient were at increased risk of loco-regional spread, then perhaps chemotherapy (or some other systemic treatment) could be proactively prescribed to improve outcomes.

There are several limitations for this study. First, the sample size was small because of strict inclusion and exclusion criteria; second, we only analyzed the post-contrast post-SBRT CT images because of non-availability of pre-contrast CT images at the same time point for each patient; third, this was a preliminary analysis and 5-fold cross validation was applied, and it would be better to have an independent validation cohort to confirm these findings. Nevertheless, this is a promising proof of principle and a hypothesis generating study.

## Conclusion

In this study, we showed that imaging features derived from the CT images 3 months after SBRT could prognosticate recurrence. The imaging feature-based models performed better than those based on clinical variables alone. Vascular involvement, vessel attachment and relative tumor enhancement were vascular related risk factors. Lymphadenopathy, pleural retraction and shape and texture related radiomic features, were also independent prognostic factors of survival. These features could provide recurrence related information, and would be helpful in clinical decision-making.

## Additional file

Additional file 1: Supplementary tables. (DOCX 43 kb)

## Abbreviations

AUC: Area under the receiver operating characteristic curve
CCI: Charlson comorbidity index
CI: Confidence interval
CT: Computed tomography
ECOG: Eastern Cooperative Oncology Group
GGO: Ground glass opacity
HR: Hazard ratio
ICC: Intra-class correlation coefficient
LR-RFS: Loco-regional recurrence-free survival
NSCLC: Non-small cell lung cancer
OS: Overall survival
PCA: Principle component analysis
PET-CT: Positron emission tomography - computed tomography
RECIST: Response Evaluation Criteria in Solid Tumor
RFS: Recurrence-free survival
RILI: Radiation induced lung injury
SBRT: Stereotactic body radiotherapy
SUVmax: Maximum standardized uptake value
