# Supplementary material for: CT imaging features associated with recurrence in non-small cell lung cancer patients after stereotactic body radiotherapy
Source: Radiat Oncol. 2017 Sep 25;12:158. doi: 10.1186/s13014-017-0892-y (PMC5613447; doi:10.1186/s13014-017-0892-y)
Supplement: Additional file 1: — Supplementary tables. (DOCX 43 kb) [file 13014_2017_892_MOESM1_ESM.docx]

### Supplementary materials

**Table 1.** Radiological scoring sheet of lung tumors

| Characteristics | | | Definition | Scoring definition |
| --- | --- | --- | --- | --- |
| lesion | Location | Distribution | central location: tumor located in the segmental or more proximal bronchi  peripheral location: tumor located in the subsegmental bronchi or more distal airway | 0 = central  1 = peripheral |
|  |  | Lobe location | lobe location of the tumor | 1 = right upper lobe (RUL)  2 = right middle lobe (RML)  3 = right lower lobe (RLL)  4 = left upper lobe (LUL)  5 = left lower lobe (LLL) |
|  | Size | Long axis diameter | longest diameter of the tumor |  |
|  |  | Short axis diameter | longest perpendicular diameter in the same slice |  |
|  |  | percentage of size changes | compared with the tumor size at the planning CT |  |
|  | Shape | Contour | the overall shape of roundness | 1 = round  2 = oval  3 = somewhat irregular  4 = irregular |
|  |  | Lobulation | a lobulated border was defined when a portion of a lesion’s surface showed a wavy or scalloped configuration | 1 = none  2 = lobulation number less than 3  3 = lobulation number between 4 and 6  4 = lobulation number more than 6 |
|  |  | Concavity | concave cuts | 1 = none  2 = slight concavity  3 = deep concavity |
|  | Margin | Border definition | well or ill-defined border | 1 = well defined  2 = everything else between 1 and 3  3 = poorly defined |
|  |  | Spiculation | lines radiating from the margins of the tumor | 1 = none  2 = fine spiculation  3 = coarse spiculation |
|  | Density | attenuation | Solid, part solid or non-solid | 1 = non-solid  2 = part solid  3 = solid |
|  |  | Calcification | any patterns of calcification in the tumor | 0 = no  1 = yes |
|  |  | Relative enhancement | use the artery on the same slice as the reference site * | Density of the lesion/density of the artery |
|  |  | Enhancement heterogeneity | Tumor heterogeneity after enhancement |  |
|  | Internal | Air bronchogram | tube like or branched air structure within the tumor | 0 = absence of air bronchogram  1 = presence of air bronchogram |
|  |  | Bubble-like lucency | the presence of air in the tumor at the time of diagnosis | 0 = absence of bubble-like lucency  1 = presence of bubble-like lucency |
|  |  | Vascular involvement | Vessels were dilated, distorted or encased by the tumor | 0 = absence  1 = presence |
| lung | | Fissure attachment | tumor attaches to the fissure, tumor's margin is obscured by the fissure | 0 = no  1 = yes |
|  |  | Pleural attachment | tumor attaches to the pleura other than fissure, tumor's margin is obscured by the pleura | 0 = no  1 = yes |
|  |  | Vessel attachment | tumor was attached to vessel | 0 = absence  1 = presence |
|  |  | Vascular convergence | convergence of vessels to the tumor, only applied to the peripheral tumors | 0 = no significant convergence  1 = obvious convergence |
|  |  | Thickened adjacent bronchovascular bundles | widening of adjacent bronchovascular bundle | 0 = None  1 = Normally tapering bundle leading to the nodule was observed to be distinctly widened |
|  |  | Pleural retraction | retraction of the pleura towards the tumor | 0 = absence  1 = presence |
|  |  | Focal emphysema | Focal emphysema caused by the tumor or preexisting emphysema | 1 = absence  2 = slight or moderate  3 = severe |
|  |  | Focal fibrosis | focal fibrosis caused by the tumor or preexisting fibrosis | 1 = absence  2 = slight or moderate  3 = severe |
|  |  | Nodules in primary tumor lobe | any non-calcified nodules suspected malignant or indeterminate | 0 = no  1 = yes |
|  |  | Nodules in non-tumor lobes | any non-calcified nodules suspected malignant or indeterminate | 0 = no  1 = yes |
|  |  | New or enlarging nodule in tumor lobe | Compared with the planning CT images before SBRT, whether there is any new or growing nodules in the tumor lobe | 0 = no  1 = yes |
|  |  | New or enlarging nodule in non-tumor lobe | Compared with the planning CT images before SBRT, whether there is any new or growing nodules in non-tumor lobe | 0 = no  1 = yes |
|  |  | Post-radiation inflammation | Inflammation in the radiated area | 0 = no  1 = yes |
|  |  | Post-radiation solidation | Solidation in the radiated area | 0 = no  1 = yes |
| thorax | | Lymphadenopathy | thoracic lymph nodes (hilar or mediastinal) with short axis diameter greater than 1 cm | 0 = no  1 = yes |
|  |  | Pleural effusion of tumor side | pleural effusion in the tumor side of thoracic cavity | 0 = no  1 = yes |
|  |  | Pleural effusion of non-tumor side | pleural effusion in the non-tumor side of thoracic cavity | 0 = no  1 = yes |

* As the pre-contrast CT images were not available for these patients, the density of the artery on the same slice was taken as reference.

**Table 2.** The number of recurrences that confirmed by biopsy, PET-CT or follow-up assessment

| confirmation method  recurrence number of patients | biopsy | PET-CT | follow-up CTs | Total |
| --- | --- | --- | --- | --- |
| Local recurrence | 6 | 4 | 5 | 11^*^ |
| Regional recurrence | 5 | 3 | 0 | 8 |
| Distant metastasis | 10 | 7^#^ | 3 | 19^†^ |

^*^There were 4 patients who were diagnosed as local recurrence based both on hypermetabolic on PET and growing / new nodule on follow-up CT scans. One patient was diagnosed with follow-up CT scans only, but the recurrence was 3 years after SBRT.

^#^ Brain metastasis was diagnosed by MR scan in one patient.

^†^ One patient was diagnosed as metastasis to liver based both on PET and CT images.

**Table 3** concordance between two readers on scoring of semantic features

| CT features | Kappa | intra-class correlation of coefficient (ICC) |
| --- | --- | --- |
| Location | 1 |  |
| Distribution | - |  |
| Fissure attachment | 1 |  |
| Pleural attachment | 0.85 |  |
| Contour | 0.78 |  |
| Lobulation | 0.88 |  |
| Concavity | 0.87 |  |
| Border definition | 0.89 |  |
| Spiculation | 0.95 |  |
| Attenuation |  |  |
| Air bronchogram | 0.81 |  |
| Bubble-like lucency | 0.93 |  |
| Calcification | - |  |
| Vascular convergence | 0.97 |  |
| Thickened adjacent bronchovascular bundles | 0.77 |  |
| Vascular involvement | 0.95 |  |
| Attachment to vessel | 0.86 |  |
| Pleural retraction | 0.68 |  |
| Peripheral emphysema | 0.81 |  |
| Peripheral fibrosis |  |  |
| Nodules in primary tumor lobe | 0.95 |  |
| Nodules in non-tumor lobes | 0.96 |  |
| Lymphadenopathy | 0.96 |  |
| Enhancement heterogeneity | 0.97 |  |
| Pleural effusion of tumor side | 0.95 |  |
| Pleural effusion of non-tumor side | 0.79 |  |
| Post-radiation inflammation | 1 |  |
| Post-radiation solidation | 1 |  |
| New or enlarging nodule in tumor lobe | 1 |  |
| New or enlarging nodule in non-tumor lobe | 1 |  |
| Long axial diameter |  | 0.95 (95% CI: 0.92 - 0.97) |
| Short axial diameter |  | 0.92 (95% CI: 0.87 - 0.95) |
| Percentage of size changes |  | 0.90 (95% CI: 0.83 - 0.94) |
| Relative enhancement |  | 0.71 (95% CI: 0.54 - 0.82) |

**Table 4** Significant prognostic clinical, semantic and radiomics features in univariate and multivariate analysis

| Survival | Feature category | Feature | Level | *p*-value | Hazard Ratio | | |
| --- | --- | --- | --- | --- | --- | --- | --- |
|  |  |  |  |  | Point | 95% CI | |
|  |  |  |  |  |  | Lower | Upper |
| OS | clinical | T stage^*^ (reference = 1A or 1B) | 2A or 2B | 0.03 | 2.27 | 1.08 | 4.77 |
|  |  | ECOG^*^ (reference = 0 or 1) | 2 or 3 | 0.01 | 3.24 | 1.38 | 7.61 |
|  | Semantic | Location (reference = 1-2) | 3-5 | 0.09 | 1.92 | 0.90 | 4.10 |
|  |  | Long axial diameter | | 0.01 | 1.67 | 1.17 | 2.38 |
|  |  | Short axial diameter | | 0.05 | 1.39 | 1.01 | 1.92 |
|  |  | Pleural attachment (reference = 0) | 1 | 0.07 | 1.89 | 0.95 | 3.76 |
|  |  | Border definition (reference = 1-2) | 3 | 0.01 | 2.45 | 1.21 | 4.96 |
|  |  | Vascular involvement^*^ (reference = 0) | 1 | 0.00 | 3.23 | 1.55 | 6.70 |
|  |  | Lymphadenopathy^*^(reference = 0) | 1 | 0.03 | 2.20 | 1.06 | 4.57 |
|  |  | Pleural effusion of tumor side (reference = 0) | 1 | 0.02 | 2.76 | 1.22 | 6.25 |
|  |  | Relative enhancement |  | 0.02 | 1.52 | 1.07 | 2.16 |
|  |  | Enhancement heterogeneity (reference = 1) | 2-3 | 0.04 | 2.13 | 1.02 | 4.42 |
|  | radiomics | F13 (9b_3D_Circularity) ^*^ | | <0.00 | 2.09 | 1.40 | 3.12 |
|  |  | F29 (Radius of smallest enclosing ellipse) | | 0.00 | 1.78 | 1.22 | 2.59 |
|  |  | F51 (avgLRE) | | 0.00 | 1.77 | 1.23 | 2.56 |
|  |  | F188 (Histogram KUR Layer 1) | | 0.00 | 1.58 | 1.16 | 2.17 |
|  |  | F190 (3D Wavelet decomposition. P2 L2 C9 Layer 1) | | 0.00 | 0.52 | 0.34 | 0.81 |
|  |  | F198 (3D Wavelet decomposition. P2 L2 C1 Layer 1) | | 0.00 | 0.50 | 0.32 | 0.79 |
|  |  | F214 (3D Wavelet decomposition. P1 L2 C3 Layer 1) | | 0.00 | 1.53 | 1.14 | 2.04 |
|  |  | PC1, per 1 SD increase^*^ | | 0.00 | 1.39 | 1.17 | 1.65 |
| RFS | clinical | T stage^*^ (reference = 1A or 1B) | 2A or 2B | 0.01 | 2.26 | 1.19 | 4.30 |
|  | Semantic | Location (reference = 1-2) | 3-5 | 0.07 | 1.84 | 0.96 | 3.52 |
|  |  | Long axial diameter | | 0.00 | 1.62 | 1.19 | 2.22 |
|  |  | Short axis diameter | | 0.01 | 1.45 | 1.10 | 1.90 |
|  |  | Pleural attachment (reference = 0) | 1 | 0.02 | 2.09 | 1.13 | 3.86 |
|  |  | Border definition (reference = 1-2) | 3 | 0.02 | 2.15 | 1.12 | 4.12 |
|  |  | Spiculation (reference = 1) | 2 | 0.09 | 1.82 | 0.91 | 3.65 |
|  |  |  | 3 | 0.00 | 3.80 | 1.69 | 8.54 |
|  |  | Thickened adjacent bronchovascular bundle (reference = 0) | 1 | 0.03 | 2.62 | 1.13 | 6.06 |
|  |  | Vascular involvement*(reference = 0) | 1 | 0.01 | 2.37 | 1.25 | 4.48 |
|  |  | Vessel attachment*(reference = 0) | 1 | 0.06 | 1.79 | 0.98 | 3.26 |
|  |  | Pleural retraction* (reference = 0) | 1 | 0.05 | 1.83 | 1.00 | 3.36 |
|  |  | Lymphadenopathy* (reference = 0) | 1 | 0.00 | 2.58 | 1.36 | 4.88 |
|  |  | Pleural effusion of tumor size (reference = 0) | 1 | 0.02 | 2.39 | 1.17 | 4.90 |
|  |  | Relative enhancement* |  | 0.02 | 1.52 | 1.07 | 2.16 |
|  | radiomics | F1 (Longest Diameter) | | 0.00 | 1.62 | 1.20 | 2.18 |
|  |  | F2 (Short Axis * Longest Diameter) | | 0.00 | 1.48 | 1.16 | 1.87 |
|  |  | F13 (9b_3D_Circularity) | | 0.00 | 1.71 | 1.27 | 2.31 |
|  |  | F14 (9c_3D_Compactness) | | 0.00 | 1.43 | 1.13 | 1.82 |
|  |  | F15 (9d_3D_AV_Dist_COG_To_Border) | | 0.00 | 1.55 | 1.15 | 2.08 |
|  |  | F28 (Radius of largest enclosed ellipse) | | 0.01 | 0.60 | 0.42 | 0.86 |
|  |  | F29 (Radius of smallest enclosing ellipse)^*^ | | 0.00 | 1.67 | 1.24 | 2.25 |
|  |  | F37 (Thickness) | | 0.01 | 1.47 | 1.11 | 1.94 |
|  |  | F48 (avgGLN) | | 0.01 | 1.51 | 1.12 | 2.03 |
|  |  | F51 (avgLRE) | | 0.00 | 1.66 | 1.22 | 2.27 |
|  |  | F188 (Histogram KUR Layer 1) | | 0.00 | 1.68 | 1.25 | 2.26 |
|  |  | F214 (3D Wavelet decomposition. P1 L2 C3 Layer 1)^*^ | | 0.00 | 1.61 | 1.18 | 2.19 |
|  |  | PC1^*^ | | 0.00 | 1.22 | 1.10 | 1.36 |
| LR-RFS | clinical | T stage^*^ (reference = 1A or 1B) | 2A or 2B | 0.04 | 2.03 | 1.03 | 4.00 |
|  |  | ECOG^*^ (reference = 0 or 1) | 2 or 3 | 0.02 | 2.47 | 1.12 | 5.44 |
|  | Semantic | Long axial diameter | | 0.01 | 1.61 | 1.15 | 2.27 |
|  |  | Short axial diameter | | 0.02 | 1.43 | 1.06 | 1.94 |
|  |  | border definition (reference = 1-2) | 3 | 0.01 | 2.38 | 1.22 | 4.63 |
|  |  | Speculation (reference = 1) | 3 | 0.00 | 4.39 | 1.87 | 10.32 |
|  |  | Vascular involvement* (reference = 0) | 1 | 0.00 | 3.78 | 1.92 | 7.43 |
|  |  | Lymphadenopathy* (reference = 0) | 1 | 0.02 | 2.17 | 1.12 | 4.21 |
|  |  | Pleural effusion of tumor size (reference = 0) | 1 | 0.03 | 2.25 | 1.07 | 4.72 |
|  |  | Relative enhancement |  | 0.02 | 1.48 | 1.06 | 2.06 |
|  |  | Enhancement heterogeneity (reference = 1) | 2-3 | 0.02 | 2.30 | 1.18 | 4.51 |
|  | radiomics | F1 (Longest Diameter) | | 0.01 | 1.55 | 1.14 | 2.12 |
|  |  | F2 (Short Axis * Longest Diameter) | | 0.01 | 1.46 | 1.11 | 1.93 |
|  |  | F13 (9b_3D_Circularity) ^*^ | | 0.00 | 1.94 | 1.39 | 2.72 |
|  |  | F14 (9c_3D_Compactness) | | 0.00 | 1.54 | 1.19 | 1.99 |
|  |  | F15 (9d_3D_AV_Dist_COG_To_Border) | | 0.00 | 1.63 | 1.19 | 2.23 |
|  |  | F16 (9e_3D_SD_Dist_COG_To_Border) | | 0.00 | 1.60 | 1.17 | 2.19 |
|  |  | F26 (Elliptic Fit) | | 0.00 | 0.59 | 0.41 | 0.85 |
|  |  | F29 (Radius of smallest enclosing ellipse) | | 0.00 | 1.74 | 1.24 | 2.42 |
|  |  | F37 (Thickness) | | 0.01 | 1.52 | 1.12 | 2.04 |
|  |  | F51 (avgLRE) | | 0.00 | 1.68 | 1.22 | 2.32 |
|  |  | F89 (3D Laws features L5 L5 E5 Layer 1) | | 0.01 | 1.55 | 1.12 | 2.15 |
|  |  | F90 (3D Laws features L5 L5 L5 Layer 1) | | 0.01 | 1.46 | 1.11 | 1.91 |
|  |  | F92 (3D Laws features L5 L5 S5 Layer 1) ^*^ | | 0.00 | 1.72 | 1.21 | 2.44 |
|  |  | F190 (3D Wavelet decomposition. P2 L2 C9 Layer 1) | | 0.00 | 0.56 | 0.39 | 0.82 |
|  |  | F198 (3D Wavelet decomposition. P2 L2 C1 Layer 1) | | 0.00 | 0.54 | 0.36 | 0.80 |
|  |  | PC1^*^ | | 0.00 | 1.23 | 1.10 | 1.37 |
|  |  | PC2 ^*^ | | 0.00 | 1.56 | 1.19 | 2.05 |

^*^: features that were still significant in multivariate analysis.

**Table 5** The number of eligible patients for validation analysis

| Group | OS | | RFS | | LR-RFS | |
| --- | --- | --- | --- | --- | --- | --- |
|  | n | % | n | % | n | % |
| Short term survival (≤ 24 months) | 17 | 30.4 | 32 | 54.2 | 26 | 44.1 |
| Long term survival (> 24 months) | 39 | 69.6 | 27 | 45.8 | 33 | 55.9 |
| Total | 56 | 100 | 59 | 100 | 59 | 100 |
